# Influence of Diet on Bowel Function and Abdominal Symptoms in Children and Adolescents with Hirschsprung Disease—A Multinational Patient-Reported Outcome Survey

**DOI:** 10.3390/children11091118

**Published:** 2024-09-12

**Authors:** Judith Lindert, Hannah Day, Marta de Andres Crespo, Eva Amerstorfer, Sabine Alexander, Manouk Backes, Carlotta de Filippo, Andrzej Golebiewski, Paola Midrio, Mazeena Mohideen, Anna Modrzyk, Anette Lemli, Roxana Rassouli-Kirchmeier, Marijke Pfaff-Jongman, Karolina Staszkiewicz, Lovisa Telborn, Pernilla Stenström, Karolin Holström, Martina Kohl, Joe Curry, Stavros Loukogeorgakis, Joseph R Davidson

**Affiliations:** 1 Department of Paediatric Surgery, University Rostock, Ernst-Heydemann-Str. 8, 18057 Rostock, Germany; marta.deandrescrespo1@nhs.net; 2 Department of Specialist Neonatal and Paediatric Surgery Great, Ormond Street Hospital NHS Trust, London WC1N 3JH, UK; hannah.day@gosh.nhs.uk (H.D.); joe.curry@gosh.nhs.uk (J.C.); stavros.loukogeorgakis@gosh.nhs.uk (S.L.); joseph.davidson@ucl.ac.uk (J.R.D.); 3 UCL GOSH Institute of Child Health, 30 Guilford Street, London WC1N 1EH, UK; 4 Department of Paediatric Surgery, University Hospital Graz, Auenbruggerplatz 34, 8036 Graz, Austria; eva.amerstorfer@medunigraz.at; 5 SoMA e.V.—Selbsthilfeorganisation für Betroffene von Morbus Hirschsprung und Anorektale Fehlbildungen Patient Organization, Munich, Blombergstr. 9, 81825 München, Germany; s.alexander@soma-ev.de (S.A.); annette.lemli@soma-ev.de (A.L.); 6 Department of Paediatric Surgery, Radboudumc Njjmegen, Geert Grooteplein Zuid 32, 6525 Nijmege, The Netherlands; manouk.backes@radboudumc.nl (M.B.); roxana.rassouli-kirchmeier@radboudumc.nl (R.R.-K.); 7 Italian Association of Hirschsprung’s Disease A.Mor.Hi, Via dei Castani 116, 00172 Rome, Italy; carlotta.defilippo@cnr.it; 8 Institute of Agricultural Biology and Biotechnology, National Research Council (CNR), Via Moruzzi, 1, 56124 Pisa, Italy; 9 Department of Surgery and Urology for Children and Adolescents, Medical University of Gdansk, 80-210 Gdansk, Poland; andrzej.golebiewski@gumed.edu.pl; 10 Pediatric Surgery Unit, Ca’Foncello Hospital, Piazzale dell´Ospedale 1, 31100 Treviso, Italy; paola.midrio@aulss2.veneto.it; 11 SoMA Austria—Selbsthilfeorganisation für Betroffene von Morbus Hirschsprung und Anorektale Fehlbildungen, Am-Ostrom-Park 11/7, 1220 Wien, Austria; mazeena.mohideen@soma-austria.at; 12 Department of Children’s Developmental Defects Surgery and Traumatology, Medical University of Silesia, 41-800 Zabrze, Poland; amodrzyk@szpital.zabrze.pl; 13 Dutch Patient Association, Vereniging Ziekte van Hirschsprung, Hambakenwetering 15, 5231 ‘S-Hertogenbosch, The Netherlands; marijke@hirschsprung.nl; 14 Polish Hirschsprung Facebook Group, Poland; karolina.staszkiewicz@poczta.onet.pl; 15 Department of Pediatric Surgery, Lund University, Skåne University Hospital, 22100 Lund, Sweden; lovisa.telborn@med.lu.se (L.T.); pernilla.stenstrom@med.lu.se (P.S.); 16 Swedish Patient Association-Hirschsprungs Sjukdom Patientförening, Sockervägen 25, 23253 Akarp, Sweden; 17 Paediatric Gastroenterology, Paediatric Department, University Lübeck, Ratzeburger Alle 160, 23538 Lübeck, Germany; martina.kohl-sobania@uksh.de

**Keywords:** Hirschsprung disease, nutrition, food items, probiotic use, patient-reported outcome of Hirschsprung disease, bowel functions related to Hirschsprung

## Abstract

**Introduction:** This study aimed to understand the influence of diet and nutrition items on gastrointestinal symptoms in patients with Hirschsprung Disease (HD). **Method:** An online questionnaire was created to obtain patient-reported outcomes using the multinational Holistic Care in Hirschsprung Disease Network. This was distributed in Dutch, English, German, Italian, Polish, and Swedish via patient associations. Information on demographics, the extension of disease, current diet, and the influence of food ingredients on bowel function were obtained. **Results:** In total, 563 questionnaires were answered by parents or patients themselves. The length of the aganglionic segment was short in 33%, long in 45%, total colonic aganglionosis (TCA) in 11%, and involved the small intestine in 10%. Overall, 90% reported following a mixed diet, and 31% reported taking probiotics, with twice as many patients taking probiotics in the TCA group compared to standard HD. Mealtimes and behaviours around eating were affected by 61%, while 77% had established food items that worsened symptoms, and of these, 80% stated that they had worked these items out themselves. A high-fibre diet was followed by 24% and 18% a low-fibre diet. Symptoms were reported, particularly from dairy in 30%, fruits in 39%, pulses in 54%, and sugar in 48%. **Conclusions:** This first multinational survey on diet and bowel function in HD reports an association between certain dietary items with gastrointestinal symptoms. This study can support an improved understanding of the interaction between food items and bowel function in children with HD. We suggest a multidisciplinary approach to balance dietary exclusions and support adequate growth, preventing nutrition deficiencies and enhancing quality of life.

## 1. Introduction

Hirschsprung Disease (HD) involves a congenital absence of enteric ganglion cells, resulting in functional distal bowel obstruction. Surgical management involves the resection of the affected aganglionic segment and pull-through of the healthy bowel, for which there are several well-established techniques with similar long-term outcomes [1]. Even after optimal surgical management, many patients may suffer long-term problems with defecation, mainly soiling and constipation, as well as Hirschsprung-associated Enterocolitis (HAEC) [2,3,4,5]. These bowel symptoms need to be addressed during follow-up to limit their impact on the quality of life (QoL) for patients and their families [2,4,5]. A recent study of parents of children with HD demonstrated a strong influence of diet on bowel function, with up to 70% of patients reporting diet-related symptoms [3,6]. Furthermore, the latest ERNICA guidelines recommend attention to dietary modifications as an integral part of the postoperative pathway of care [7]. Specialist information on nutrition and bowel function were also among the key items identified by parents [8].

Nutritional intake during childhood determines physical, cognitive, and emotional development and is integral to social interactions. Adequate nutrition is also critical for proper neurodevelopment and the establishment of a fully functional immune system [9]. This is particularly important for patients with HD, where ongoing problems with bowel management can affect the time for regular socialising and lead to increased school absenteeism and poor academic performance [10]. These symptoms undoubtedly affect the QoL of patients and their parents.

In individuals with chronic constipation who do not have HD, medications such as stool softeners are generally the first-line recommendation [11]. Nevertheless, dietary measures, such as increasing fluid intake, adjusting fibre intake and avoiding cow’s milk, are common practices in the management of paediatric constipation [12]. These recommendations are often replicated for the treatment of HD. To our knowledge, no international study has been conducted to evaluate whether specific dietary adjustments may affect bowel function in patients with HD.

This study uses patient-reported outcomes to describe the influence of diet on bowel functions and symptoms in children and adults with HD, as reported by patients/carers, using a multilingual, international questionnaire.

## 2. Materials and Methods

### 2.1. Study Design and Design of Online Questionnaire

An online questionnaire on dietary practice, food items, and bowel function was developed in English by the multinational OASIS Holistic Care in HD Network in preparation for the OASIS Holistic Care in Hirschsprung Disease Summer Symposium 2023. The questionnaire was developed by an international stakeholder group, including surgeons, physicians, dietitians and patient representatives, in a series of virtual consensus meetings. The questionnaire was translated into Dutch (R R-K, MB), German (EA, JL), Italian (CdF, PM), Polish (AM), and Swedish (LT, PS) by native speakers with a medical background and programmed into REDcap (JD, MC). The survey was disseminated through patient associations via newsletters, internet homepages, and social media groups in Austria, Germany, Italy, Netherlands, Poland, the United Kingdom, and Sweden (AL, SA, CdF, KS, MP-J, JD, MM). The questionnaire was open between July and October 2023, and patients could choose to complete the questionnaire in any language (e.g., anyone could complete it in English, etc.). The full questionnaire is available as Supplementary Material.

### 2.2. Definitions Used in the Questionnaire

We defined the level of disease as follows for this questionnaire:

Short disease (involving just the rectum), long disease (involving more than the rectum but not the whole colon), total colonic aganglionosis (the whole colon), and small intestine (the whole colon as well as a length of the small bowel). Throughout the manuscript, comparisons are made between patients with and without their colon in situ (i.e., rectosigmoid + extended segment vs. total colonic aganglionosis with/without small intestinal involvement).

The questionnaire included questions on 10 specific food groups to investigate effects on bowel function; specifically, the following 10 items were enquired after: 1, I pass stools more often|2, My stools are more liquid|3, I pass stools less often|4, My stools are harder|5, I have more problems with soiling (staining in the underwear)|6, I have more accidents with stools|7, I have more bloating|8, I have more cramping pains|9, I have more issues with flatulence|10, No symptoms|11, Other (please specify).

The informants were parents of children with HD or HD patients themselves to obtain patient-reported outcomes (PROs). Information on demographics, disease severity, current diet, and the influence of food components on bowel function was answered by the families directly. Patients who had toilet-trained children and were not managing an enterostomy were asked to complete the Rintala Bowel Function Score (BFS).

### 2.3. Statistical Analysis

The answers were captured on REDcap and subsequently analysed using SPSS 26.0 and Prism 9.0 (GraphPad).

Data are presented either as percentages (*n*(%)) or as the median with an associated interquartile range (median [IQR]). Appropriate statistical tests were performed for categorical (Chi-square) or continuous (Mann–Whitney U) variables. Patients with incomplete questionnaires were included as long as the disease characteristics were present; therefore, throughout the manuscript, denominators for different aspects of the survey differ.

### 2.4. Ethics

The study was approved as a multinational survey study by the Ethics Committee of the University of Rostock in July 2023 (July/23: A 2023-0123). Participants answered anonymously, and no identification could be tracked.

## 3. Results

### 3.1. Characteristics of Patients Participating in the OASIS Nutrition Survey

Overall, 563 patients from seven countries responded to the questionnaire (Table 1). Almost 1/3 of respondents answered in German (Germany/Austria), while just over one quarter answered in Italian. While 70–80% of a typical HD population would be expected to have short-segment disease—these accounted for just 33% of our study cohort—a more severe phenotype was suggested in those who were active in patient support groups. Approximately one in eleven patients managed with an enterostomy.

Bowel function outcome was assessed in patients who were toilet-trained and did not have an enterostomy (*n* = 290). Table 2 depicts the results with completed scores by segment length. We noted, surprisingly, that in this cohort, patients with shorter segment HD did not have superior functional outcomes when assessed with the Rintala Bowel Function Score (BFS; overall BFS out of 20—colon in situ vs. removed: 15(12–17) vs. 15(13–17); Mann–Whitney U: *p* = 0.311). It is important also to notice that only around half of the patients were assessed with the BFS; shorter segment patients were more likely to not have been toilet trained yet (154/420 (37%) vs. 18/114 (16%); *p* < 0.0001) and longer segment patients were not assessed as they were more likely to have stoma (27/114 (23%) vs. 24/420 (6%); *p* < 0.0001)

### 3.2. Dietary Characteristics Were Assessed in Children over the Age of 2 Years

#### 3.2.1. Overall Diet

Ninety percent of respondents (449/499) followed a mixed diet, 2% stated they were vegetarian/vegan, and 7% followed a special diet with specific exclusions (for example, dairy, gluten, FODMAP, or sugars; see Table 3). While approximately half of the respondents did not pay specific attention to their dietary fibre intake, patients with a shorter segment were much more likely to opt for a high-fibre diet, while patients with total colonic disease tended to opt for lower fibre intake (*p* < 0.0001 for both).

Overall, 341/560 (61%) of patients/families reported that their HD diagnosis had altered the way they chose to eat, and 433/562 (77%) had identified specific food items or ingredients that affected their symptoms, both of which were more prevalent in patients with TCA and small bowel aganglionosis (*p* < 0.001 for both); 104 (24%) concluded this following a discussion with a healthcare professional, most commonly their surgeon (*n* = 72) or a dietitian (*n* = 53). In total, 80% (345/433) had self-identified problematic foods.

Probiotics were used by 31% of patients (177/562), and the rate of use was higher in patients with total colonic aganglionosis (TCA) and TCA with small intestinal disease (46%) compared to those with part of the colon in situ (26%; *p* < 0.0001); see Table 3. Among patients who knew the composition of probiotics, 64% reported taking multi-strain products such as Omniflora^®^: *Lactobacillus gasseri* and *Bifidobacterium longum*, VSL3^®^: *S. thermophilus* BT01, *B. breve* BB02, *B. animalis* subsp. lactis BL03, *B. animalis* subsp. lactis BI04, *L. acidophilus* BA05, *L. plantarum* BP06, *L. paracasei* BP07, *L. delbrueckii* subsp. bulgaricus BD08 or Vivomixx^®^: * *Streptococcus thermophilus* DSM24731^®^, *Bifidobacterium breve* DSM24732^®^, *Bifidobacterium longum* DSM24736^®^, *Bifidobacterium infantis* DSM24737^®^, *Lactobacillus acidophilus* DSM24735^®^, *Lactobacillus plantarum* DSM24730^®^, *Lactobacillus paracasei* DSM24733^®^, and *Lactobacillus delbrueckii* ssp. *bulgaricus* DSM24734^®^ DSM24734^®^. Interestingly, although most patients took multi-strain products, among Italian patients, most patients opted to take *Lactobacillus monoculture* products, such as *L. rhamnosus* and *L. reuteri*.

#### 3.2.2. Food-Related Dietary Symptoms

**Figure 1:**
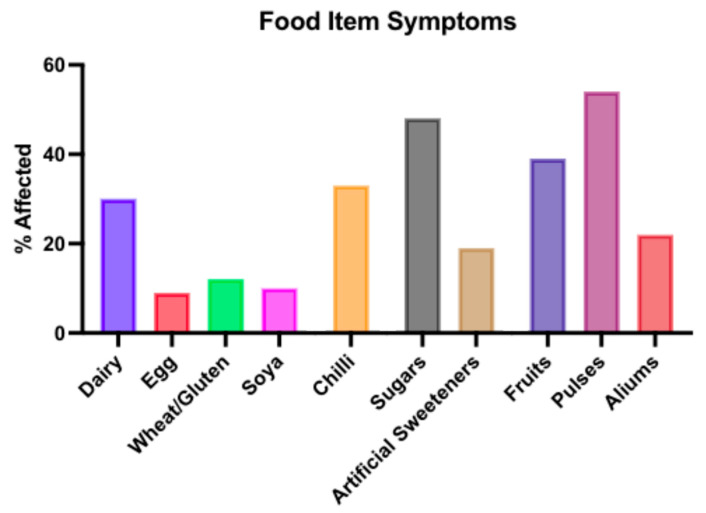
Overall onset of adverse bowel symptoms noted by patients and their families according to food items.

We asked enclosed questions on these 10 separate food items, and the results are displayed in Table 4. Symptoms were more commonly seen in patients without their colon in situ for all except eggs and high-sugar-containing foods. High-sugar foods were related to decreased stool frequency—(25/474) 5%; harder consistency—(41/474) 9%; increased soiling—(58/474) 12%; and more faecal accidents—(21/474) 4%. The different symptoms relating to respective food items are displayed in Table 4, while the most frequent symptoms associated with the three most problematic foods (pulses, high-sugar foods, and fruit) are shown in Table 5. A comparative overview of the proportion of patients reporting any symptoms according to food items is shown graphically in Figure 1.

## 4. Discussion

This multinational questionnaire was answered by over 500 patients (*n* = 563) from seven European countries: the largest patient-reported study on dietary aspects of gastrointestinal symptoms in HD. Overall, 3 in 5 patients reported that HD affected the items they chose to eat, while three-quarters established food items that worsened symptoms, and, of these, 80% stated that they had worked these items out themselves without any support from healthcare professionals.

This questionnaire addresses an important issue for families, which was specifically highlighted in qualitative focus group discussions, where families stated the key influence that diet has on their child’s bowel function [3]. There are currently no dietary recommendations for patients, particularly children, diagnosed with HD. Specific recommendations for those with increased colonic transit include a constipating diet with bulking agents. In general, constipation, or, more likely, outlet obstruction, is reported to affect up to 80% of children with HD [13,14]. Healthy young children would open their bowel once daily with soft stool and usually take a low fibre diet [15].

### 4.1. Functional Outcomes and Impact of Specific Food Items

The patient cohort was assessed using Rintala BFS to make some objective measures of functional status. Interestingly, by dividing the cohort by age and segment length, we could see that the documented population is not representative of the overall HD population. Specifically, only one-third of patients had rectosigmoid disease (compared to the 80% expected [6]), and the patients with rectosigmoid disease did not have better bowel outcomes than those with longer segments, as has been reported elsewhere [2]. However, in spite of worse-than-expected function in patients with short segments, they experienced a much-reduced impact from the consumption of various food items, took fewer probiotics, and fewer patients declared that HD affected how they chose to eat.

Diet has also been reported by parents to be the dominant influence on their child’s bowel function [6]. Among our cohort, gastrointestinal symptoms were most commonly noted in relation to pulses (54%), sugars (48%), fruits (39%), and dairy (30%). Analysing the type of adverse bowel symptoms that were reported, the most common was diarrhoea, which was the most prevalent for dairy (51%) and sugars (36%).

Comparatively fewer patients reported symptoms for egg (9%), wheat/gluten (12%), and soya (10%), which is in line with the results from a previous study in a Swedish setting [6]. 

Foods high in refined sugar provoked symptoms in 48% of patients compared to 19% for artificial sweeteners. A similar pattern has been shown in patients with inflammatory bowel disease (IBD), where 23% reported “Sugary/sweet food” to worsen their gastrointestinal symptoms, while only 4% specified artificial sweeteners to trigger their symptoms [16,17]. However, research into the effect of artificial sweeteners on gastrointestinal symptomatology is scarce and has produced conflicting results [17,18]. Studies in irritable bowel syndrome and inflammatory bowel disease suggest an impact secondary to changes in gastrointestinal motility and intestinal microbiota, especially induced by polyols [17,18].

Pulses were the food item most likely to cause gastrointestinal symptoms, mainly due to bloating and flatulence. Foods rich in fermentable residues, such as pulses, have also been suggested to increase intestinal gas production in healthy children [19]. However, in a study on healthy children, only 24% reported any gastrointestinal symptoms [19] compared to over 50% of children with HD in this study—suggesting an additional disease-specific effect. Previous research has shown that fruits, foods high in fat content, grains including bread and cereal, vegetables, and dairy are commonly excluded to reduce gastrointestinal symptoms [20]. Speculatively, the difference in symptom generation may be related to differences in intestinal microbiota or motility reactions to food between healthy and HD patients [21,22,23].

A low Fermentable Oligosaccharides, Disaccharides, Monosaccharides, and Polyols (FODMAPs) diet has gained popularity for the treatment of functional abdominal pain and other functional abdominal symptoms, including bloating. This diet results in a reduction in fermentable carbohydrates (FODMAPs). Many of the foods excluded by the participants in this study were high in FODMAPs, including wheat, legumes, dairy products, fruits, alliums, and some artificial sweeteners. Wheat, legumes, and alliums are classified as fructans and galacto-oligosaccharies (GOSs). These FODMAPs are fermentable by the microbiota in the colon [24,25] and, due to the limited available colon in HD, may further exacerbate symptoms. A diet high in FODMAPs may be important to consider in patients with ileostomies, as it has been shown to increase fluid output and decrease absorption [26].

The reported findings of interaction between food items and bowel function can help in counselling families to ameliorate adverse dietary symptoms; however, we believe that appropriate and supervised exclusion should be considered to enable varied nutritional intake to encourage appropriate growth and development. As there are no guidelines for specific dietary advice for people with HD, the results show that many patients and carers may modify their diet in response to symptoms perceived to be associated with specific foods. Reducing fructose and lactose in patients with HD has previously been shown to reduce symptoms of faecal incontinence [23]. Similar results were seen in our study’s families, who reported reducing intake because of loose stools.

Overall, this work suggests that there are clear food-related symptoms that occur in a large percentage of patients with HD. This raises the possibility that soiling and accidents that are noted in children may be, in fact, related to dietary intake and may trigger investigations post-pull-through, such as GI endoscopy and rectal biopsy. Dietary changes should be made under the guidance of healthcare professionals, such as dietitians, who can make personalised recommendations based on an individual’s specific needs and symptoms. Each person with HD may have a unique response to foods, so tailoring dietary changes is essential to optimise symptom management and overall health. Symptoms and dietary changes have been shown to increase stress and feelings of exhaustion [3]. Research in this area may provide further insight into effective dietary strategies for people with HD, which may help patients feel more supported in their condition.

### 4.2. Microbiome and Probiotics

In addition to dietary impacts on symptoms, this study also surveyed the use of probiotics taken by patients. In this study, 35% of the respondents used probiotics with Bifidobacteria and Lactobacillus. We further noted that patients with total colonic HD were twice as likely to use probiotics compared to patients who still had part of their colon in situ. The microbiome plays a crucial role in digestion, and it is influenced by geographical food composition and altered by the removal of a certain length of the colon or entire colon and perioperative and intermittent antibiotic use. Significant differences in the gut microbiota between healthy individuals and children with functional constipation have been described [27]. The microbiome is potentially linked to intestinal motility. Further research showed that probiotics not only significantly diminished the incidence but also decreased the severity of HAEC [28]. However, the mechanisms by which pre-, pro-, and symbiotics may reduce inflammation and their particular role in HD are not fully understood [28,29,30].

### 4.3. Differences Due to the Length of Hirschpsrung Disease Segment

We note, in this study, that patients with short-segment disease reported symptoms from specific food items less frequently than those with TCA. This was the case for all food items surveyed, with the exception of egg (which was generally low across all segment lengths) and refined sugars (around 50% across different levels of disease). Also, patients with TCA reported a higher rate of impact on their social life for the BFS, which may, in part, be due to the significantly higher dietary impact reported here. Knowledge of the resected part and remaining bowel is crucial for the medical team to anticipate bowel function and best advise parents. Often, the level of disease is judged by the removed segment of the bowel, although we suggest to rather focus on the remaining segments, which determine the function. Unfortunately, there is not yet a universally agreed consensus on categories for the length of the HD segment, and thus, a comparison remains challenging.

### 4.4. Holistic Nutrition Counselling and Specialist Dietician Contribution

**Figure 2:**
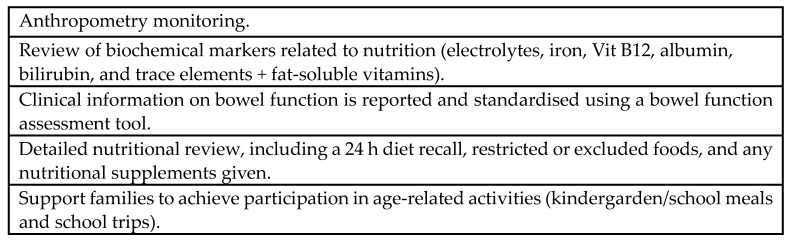
Checklist role of dietician.

In this patient-reported study, we demonstrate that in the majority of instances, patients and families had established the effects of food items themselves, often making changes and exclusions to their diet in the absence of any professional supervision. Our research highlights foods that potentially exacerbate or lead to negative bowel function symptoms; however, clearly, it cannot be determined whether exclusion could alleviate these symptoms and to what extent. We also need to carefully consider the nutritional needs of the growing child, so dietary changes, particularly exclusions, would benefit from appropriate assessment by dieticians. The role of a dietitian in the multidisciplinary Hirschsprung Disease team may include nutritional advice as well as the clinical observation and monitoring of dietary changes; see Figure 2.

Malnutrition has been recognised in children with Hirschsprung Disease, and this was particularly the case if surgery was performed after the age of 3 years [8]. At present, we cannot reliably attribute poor nutritional status to failure to thrive due to pre-existing Hirschsprung’s Disease or to dietary factors.

### 4.5. Strengths and Limitations

This is the largest cohort of patient-reported data on diet and bowel function in patients with HD. Information was collected across different age groups from seven European countries with a heterogeneous cultural dietary profile. Patient representatives were involved throughout, which has been shown to improve the quality and relevance of research [31]. We did not collect any information about our participants’ medical history (i.e., reasons for stoma formation, the timing of pull-through) and solely relied on patient/caregiver-declared information (i.e., segment length). We were not able to compare different pull-through techniques. Our study is further limited by two distinct biases: firstly, outreach through patient associations may only reach involved families—potentially emphasising an access bias known to exist across demographic differences. Secondly, we anticipate a reporting bias from those patients with more adverse symptoms (themselves more likely to be involved in patient support organisations); this is suggested by the under-representation of patients with short-segment disease and comparatively worse BFS scores in these patients than might be expected.

## 5. Conclusions

This multinational survey provides important insights into the experience of food-related clinical symptoms in patients with HD. Eggs, wheat, and soya were not reported to evoke adverse symptoms more than expected, while pulses, sugars, and fruits seem to result in adverse bowel functions in a large proportion of patients. Overall, the majority of responders reported that their diet influences bowel function and tried to limit this impact by selecting or avoiding specific food items on their own. Food counselling, involving dietitians to ensure appropriate dietary intake to support development and growth, should be delivered with the awareness that there is still very limited evidence highlighting specific foods and their impact on bowel function in patients with HD.

## Figures and Tables

**Table 1:** Patient demographics (*n* (%) or median [IQR]).

	Overall	German	English	Italian	Netherlands	Polish	Swedish
Number	563	181	29	145	101	80	27
Age, y Med (IQR)	6	6	5	7	10	4	6
	(3–11)	(3–10)	(2–13)	(3–11)	(4–20)	(2–7)	(3–10)
Segment, *n* (%)							
*Rectosigmoid*	188 (33)	48 (27)	14 (48)	66 (46)	34 (34)	20 (25)	6 (22)
*Long segment*	252 (45)	92 (51)	9 (31)	47 (32)	51 (50)	38 (48)	15 (56)
*Total colonic*	63 (11)	17 (9)	4 (14)	47 (32)	7 (7)	38 (48)	3 (11)
*Small intestine*	59 (10)	23 (13)	2 (7)	15 (10)	9 (9)	17 (21)	3 (11)
*Not declared*	1 (0.2)	1 (0.5)	0	0	0	0	0
Stoma, *n* (%), completed y/*n*	50/562 (8.9)	12/181 (6.6)	1/29 (3.4)	5/144 (3.5)	4/101 (3.9)	23/80 (23.8)	5/27 (18.5)
Toilet-trained (without a stoma, completed y/*n*)	276/447 (61.7)	84/150 (56%)	12/23 (52.2)	97/126 (76.9)	44/73 (60.3)	33/54 (61.1)	7/21 (33.3)
ACE, *n*(%), completed y/*n*	16/56 (28.6)	3/180 (1.7)	2/29 (6.9)	3/144 (2.1)	4/101 (3.9)	2/79 (2.5)	2/27 (7.4)
TAI, *n* (%), completed y/*n*	129/561 (22.9)	50/181 (27.6)	5/29 (17.2)	21/144 (14.5)	34/101 (33.6)	6/80 (7.5)	13/27 (48.1)
Tube-feeding, *n* (%), completed y/*n*	11/561 (1.9)	3/181 (1.6)	0/29	2/143 (1.4)	4/101 (3.9)	2/80 (2.5)	0/27
Parenteral feeding, *n* (%), completed y/*n*	5/561 (0.9)	0	0	1/143 (0.7)	0	4/80 (5%)	0

ACE: antegrade continence enema, TAI: transanal irrigation.

**Table 2:** Patient-reported bowel function according to the level of Hirschsprung Disease.

		Hirschsprung Disease—Colon Partially In Situ		Hirschsprung Disease—Colon Fully Removed	
		Short Disease (*n* = 107)	Long Disease (*n* = 119)	TCA (*n* = 33)	Small Intestine (*n* = 31)
**Overall Score** **(Median [IQR])**		14 [12–17]	15 [12–17]	14 [12–16]	15 [13–17]
**Are you aware of the feeling when you need to pass stool?**	Always	32 (30%)	41 (34%)	17 (52%)	16 (52%)
	Most of the time	41 (38%)	40 (34%)	11 (33%)	9 (29%)
	Often uncertain	24 (22%)	30 (25%)	3 (9%)	4 (13%)
	No awareness	10 (9%)	8 (7%)	2 (6%)	2 (6%)
**Are you able to hold back when you need to pass stools?**	Always	50 (47%)	61 (51%)	15 (45%)	20 (65%)
	Occasional problems, less than once per week	36 (34%)	35 (29%)	12 (36%)	8 (26%)
	Problems holding in stool every week	12 (11%)	12 (10%)	4 (12%)	2 (6%)
	No control over bowels, problems every day	9 (8%)	11 (9%)	2 (6%)	1 (3%)
**How often do you pass stool?**	Less than once every 2 days	7 (7%)	8 (7%)	0	0
	Every 2 days	16 (15%)	12 (10%)	0	3 (10%)
	Once per day	55 (51%)	40 (34%)	1 (3%)	2 (6%)
	Twice per day	16 (15%)	31 (26%)	4 (12%)	1 (3%)
	More than twice per day	13 (12%)	28 (24%)	28 (85%)	25 (81%)
**How often do you have issues with soiling or staining of the underwear?**	Never have issues with soiling	22 (21%)	26 (22%)	6 (18%)	11 (35%)
	Soiling less than once a week, only rarely needing a change in underwear	43 (40%)	44 (37%)	14 (42%)	10 (32%)
	Soiling every week, often requiring a change in underwear	29 (27%)	29 (24%)	7 (21%)	6 (19%)
	Soiling all the time, using protective aids	13 (12%)	20 (17%)	6 (18%)	4 (13%)
**How often do you have accidents with stools in the underwear?**	Never	45 (42%)	52 (44%)	11 (33%)	21 (68%)
	Rarely, less than once per week	37 (35%)	44 (37%)	15 (45%)	6 (19%)
	Weekly, wearing protective aids	12 (11%)	10 (8%)	2 (6%)	2 (6%)
	Daily, wearing protective aids day and night	13 (12%)	13 (11%)	5 (15%)	2 (6%)
**Do you suffer from constipation?**	No	60 (56%)	69 (58%)	29 (88%)	20 (65%)
	Constipation managed with diet	17 (16%)	15 (13%)	2 (6%)	4 (13%)
	Constipation managed with medication	20 (19%)	21 (18%)	2 (6%)	6 (19%)
	Constipation managed with enemas	10 (9%)	14 (12%)	0	1 (3%)
**What is the social impact of your bowel function?**	No impact on social life	40 (37%)	46 (39%)	6 (18%)	8 (26%)
	Some impact	48 (45%)	54 (45%)	21 (64%)	16 (52%)
	Problems that limit social activities	12 (11%)	15 (13%)	5 (15%)	2 (6%)
	Major social or psychological issues related to bowel function	7 (7%)	4 (3%)	1 (3%)	5 (16%)

**Table 3:** Dietary modification in patients ≥ 2 years (note that an age filter ≥ 2 years and age/segment blanks were removed).

	*n* = 499	Hirschsprung Disease—Colon Partially In Situ		Hirschsprung Disease—Colon Fully Removed		
		Short Disease (*n* = 167)	Long Disease (*n* = 219)	TCA (*n* = 62)	Small Intestine (*n* = 51)	*p*-Value (Chi-Square)
General diet	Mixed diet	150 (90%)	198 (90%)	55 (89%)	43 (84%)	0.300
	Pescetarian	3 (2%)	1 (0.5%)	0	0	-
	Vegetarian	2 (1%)	5 (2%)	0	2 (4%)	-
	Vegan	1 (1%)	1 (0.5%)	0	0	-
	Special/exclusion	11 (7%)	14 (6%)	7 (11%)	5 (10%)	0.239
	Complete tube feeding	0	1	0	0	-
	*n*/A (nil enteral)	0	0	0	1	-
Dietary fibre	Deliberately high	59 (35%)	66 (30%)	7 (11%)	8 (16%)	<0.0001
	Neither	81 (49%)	115 (53%)	32 (52%)	27 (53%)	
	Deliberately low	27 (16%)	38 (17%)	23 (37%)	16 (31%)	<0.0001
Probiotic use		46/187 (25%)	70/252 (28%)	33/63 (52%)	24/60 (40%)	<0.0001
Hirschsprung affects eating and mealtimes		94/186 (51%)	151/252 (60%)	49/61 (80%)	46/60 (77%)	<0.0001
Specific ingredients/items affect symptoms		115/186 (62%)	172/252 (68%)	46/61 (75%)	49/60 (82%)	0.008

**Table 4:** Food items causing gastro-intestinal symptoms in relation to the type of Hirschsprung Disease. Patients who had not tried a particular food item were not included.

Food Item	Total	Short Disease	Long Disease	TCA	Small Intestine	*p*-Value (Chi-Square)
Dairy (cows milk), *n* (%)	152/509 (30)	39/172 (23)	17/219 (8)	22/59 (37)	25/53 (47)	<0.0001
Eggs, *n* (%)	42/488 (9)	12/164 (7)	17/219 (8)	2/55 (4)	11/52 (21)	0.170
Wheat, *n* (%)	61/511 (12)	13/171 (8)	25/226 (11)	13/37 (23)	10/57 (18)	0.0004
Soya, *n* (%)	29/286 (10)	3/95 (3)	13/127 (10)	9/28 (32)	4/36 (11)	0.0043
Pulses (beans, peas, lentils), *n* (%)	220/409 (54)	63/141 (45)	101/188 (54)	30/45 (67)	26/35 (74)	0.0012
Alliums (onions, garlic), *n* (%)	90/418 (22)	19/132 (14)	37/191 (19)	13/51 (25)	21/44 (48)	0.0003
Chilli, *n* (%)	69/212 (33)	14/63 (22)	28/99 (28)	10/21 (48)	17/29 (59)	0.0005
Fruits (Top 3: apples, grapes, bananas), *n* (%)	208/531 (39)	54/175 (31)	92/244 (38)	32/62 (52)	30/51 (59)	0.0001
High-sugar foods, *n* (%)	227/474 (48)	69/161 (43)	104/211 (49)	29/56 (52)	25/46 (54)	0.265
Artificial sweeteners, *n* (%)	39/209 (19)	10/70 (14)	18/90 (20)	5/22 (23)	6/27 (22)	0.0063

**Table 5:** Food items and evoked symptoms.

Food Item (Number Affected)	Symptom (Top 3)	Prevalence of Symptoms (%)
Pulses (*n* = 220)	Flatulence	52
	Bloating	50
	Cramping	30
High-sugar foods (*n* = 227)	Bloating	37
	More liquid stools	34
	Increased frequency	33
Fruits (apples, grapes, bananas, *n* = 208)	More liquid	41
	Increased frequency	34
	Bloating	26

## Data Availability

The data presented in this study are available on request from the corresponding authors. The data are recorded in an anonymous Redcap Database due to privacy or ethical restrictions.

## Supplementary Materials

The following supporting information can be downloaded at https://www.mdpi.com/article/10.3390/children11091118/s1. Table S1: Nutrition questionnaire, English version.

## Abbreviations

ACE	Antegrade continence enema
BFS	Bowel Function Score
FODMAP	Fermentable Oligosaccharides, Disaccharides, Monosaccharides and Polyols
HD	Hirschsprung Disease
IBD	Inflammatory bowel disease
QoL	Quality of life
TAI	Transanal irrigation
TCA	Total colonic aganglionosis
