# Supplementary material for: Influence of Diet on Bowel Function and Abdominal Symptoms in Children and Adolescents with Hirschsprung Disease—A Multinational Patient-Reported Outcome Survey
Source: Children (Basel). 2024 Sep 12;11(9):1118. doi: 10.3390/children11091118 (PMC11429589; doi:10.3390/children11091118)
Supplement: Supplementary file 1 [file children-11-01118-s001.zip › children-3092343-supplementary.pdf]

HSCR Nutrition Questionnaire (English)

This survey is for patients with Hirschsprung disease or their carers. It asks about bowel function and diet.

Thank you for completing the questionnaire!

Select a language	◯ English<br>◯ Deutsch<br>◯ Polski<br>◯ Italiano<br>◯ Nederlands<br>◯ Svenska
I am a	◯ Patient with Hirschsprung's<br>◯ Parent/Carer of a child with Hirschsprung's
What is your age (in years)?	______________________
What is the age of your child (in years)?	______________________
What length of bowel is affected by Hirschsprung?	◯ Short Segment (i.e. just the rectum)<br>◯ Long Segment (i.e. more than the rectum but not the whole colon)<br>◯ Total Colonic Aganglionosis (i.e. the whole colon)<br>◯ Small Intestine (i.e. whole colon as well as a length of the small bowel)
Do you / does your child currently have a stoma?	◯ Yes<br>◯ No
This stoma is:	◯ Ileostomy (in the small bowel)<br>◯ Colostomy (in the colon)<br>◯ Not sure
Has your child toilet trained?	◯ Yes<br>◯ No
Do you / does your child currently have an ACE (antegrade colonic enema)?	◯ Yes<br>◯ No
Do you / does your child currently use a transanal irrigation system (e.g. QuFora, Peristeen, Navina)?	◯ Yes<br>◯ No
Do you / does your child currently have a feeding tube?	◯ Yes<br>◯ No
What sort of feeding tube do you use?	◯ Nasogastric tube<br>◯ Nasojejunal tube<br>◯ Gastrostomy (i.e. PEG or Mickey/Mini Button)<br>◯ Gastrostomy with jejunal extension (i.e. PEG-J, Mickey-J or G-JET)<br>◯ Other (please state
What sort of feeding tube do you use?	______________________

What type of feed do you give via the feeding tube?	◯ Blended food (this means real food blended to an appropriate consistency and delivered via the tube)<br>◯ Commercial milk feed (please state name/names of feeds)<br>◯ Mix of Blended food and Commercial feed (please state name of feed)
What commercial milk feed(s) do you use with your feeding tube?	______________________________
Are you aware of the feeling when you need to pass stools?	◯ Always aware<br>◯ Most of the time<br>◯ Often uncertain<br>◯ No awareness
Are you able to hold back when you need to pass stools?	◯ Always able<br>◯ Occasional problems holding in stool, less than once per week<br>◯ Problems holding in stool every week<br>◯ No control over bowels, problems every day
How often do you pass stool	◯ Less than once every 2 days<br>◯ Every 2 days<br>◯ Once per day<br>◯ Twice per day<br>◯ More than twice per day
How often do you have issues with soiling or staining of the underwear	◯ Never have issues with soiling<br>◯ Soiling less than once a week, only rarely needing a change of underwear<br>◯ Soiling every week, often requiring a change of underwear<br>◯ Soiling all the time, using protective aids (i.e. pads or diapers)
How often do you have accidents with stools in the underwear?	◯ Never<br>◯ Rarely, less than once per week<br>◯ Weekly, wearing protective aids<br>◯ Daily, wearing protective aids day and night
Do you suffer from constipation	◯ No constipation at all<br>◯ Constipation managed with diet<br>◯ Constipation managed with medication<br>◯ Constipation managed with enemas
Do you also take oral medication to help with this?	◯ Yes<br>◯ No
Which medication do you take for constipation?	______________________________
What is the social impact of your bowel function	◯ No impact on social life<br>◯ Some impact (i.e. bad smells or need to get to toilet quickly)<br>◯ Problems that limit social activities<br>◯ Major social or psychological issues related to bowel function

Do you ever suffer from bloating or cramping in the abdomen?	◯ Never any symptoms<br>◯ Rarely affected by bloating or cramping<br>◯ Problems with bloating or cramping most days<br>◯ Problems with bloating or cramping multiple times per day
Do you suffer from problematic flatulence (farts)?	◯ No problems at all<br>◯ Occasionally farts smell bad or I am unaware of passing wind<br>◯ Often farts smell bad or I pass wind without realising<br>◯ Never aware when passing wind and farts smell all the time
Is there anything else about your bowel function that you think would be important to tell us?	__________________________________

We would like to ask some questions about your diet and how it might affect your Hirschsprung's, please note if you are answering as a carer - these questions relate to the diet of your child

Which statement best describes your diet?	◯ I eat a mixed diet including meat and fish<br>◯ I eat a mixed diet, I avoid meat but include fish (pescatarian)<br>◯ I follow a vegetarian diet (no meat or fish)<br>◯ I follow a vegan diet (no animal products)<br>◯ Other (please specify)
Could you explain more about the diet you follow?	__________________________________
Regarding your dietary fibre intake:	◯ I deliberately eat a high-fibre diet<br>◯ I deliberately eat a low-fibre diet<br>◯ I don't pay attention to the fibre in my diet
Do you feel that Hirschsprung disease affects the way you choose to eat (i.e. diet, meal times etc.)	◯ Yes<br>◯ No
Could you briefly explain what you feel is affected?	__________________________________
Have you established that there are any food items or ingredients that make your symptoms worse?	◯ Yes<br>◯ No
How did you come to learn this food made symptoms worse? (Tick all that apply)	☐ Hospital Doctor / Surgeon advice<br>☐ GP / Family Doctor advice<br>☐ Specialist Nurse advice<br>☐ Specialist Dietician / Nutritionist advice<br>☐ Other patient / parent advice<br>☐ Worked out by myself<br>☐ Other (please specify below)
Please specify any other sources you found helpful in working out diet related changes to your symptoms:	__________________________________

Does Cow's Milk cause you symptoms (including cheese or yoghurt)?	◯ Yes<br>◯ No<br>◯ Not sure as I have never tried this
Are your issues specific to:	◯ Cow's Milk Protein<br>◯ Lactose<br>◯ Not Sure<br>◯ Other (please specify)
Please specify	__________________________
Which symptoms do you find are worse when you have cow's milk (tick all that apply)?	☐ I pass stool more often<br>☐ My stools are more liquid<br>☐ I pass stool less often<br>☐ My stools are more hard<br>☐ I have more problems with soiling (staining in the underwear)<br>☐ I have more accidents with stools<br>☐ I have more bloating<br>☐ I have more cramping pains<br>☐ I have more issues with flatulence<br>☐ No symptoms<br>☐ Other (please specify)
Which other symptoms do you have with Cow's Milk?	__________________________
Does egg cause you symptoms?	◯ Yes<br>◯ No<br>◯ Not sure as I have never tried this
Which symptoms do you find are worse when you have egg (tick all that apply)?	☐ I pass stool more often<br>☐ My stools are more liquid<br>☐ I pass stool less often<br>☐ My stools are more hard<br>☐ I have more problems with soiling (staining in the underwear)<br>☐ I have more accidents with stools<br>☐ I have more bloating<br>☐ I have more cramping pains<br>☐ I have more issues with flatulence<br>☐ No symptoms<br>☐ Other (please specify)
Which other symptoms do you have with egg?	__________________________
Does wheat or gluten cause you symptoms?	◯ Yes<br>◯ No<br>◯ Not sure as I have never tried this
Do you specifically have issues with	◯ Wheat only<br>◯ All grains containing gluten<br>◯ Not sure<br>◯ Other (please specify)
Please specify:	__________________________

Which symptoms do you find are worse when you have wheat or gluten (tick all that apply)?	☐ I pass stool more often<br>☐ My stools are more liquid<br>☐ I pass stool less often<br>☐ My stools are more hard<br>☐ I have more problems with soiling (staining in the underwear)<br>☐ I have more accidents with stools<br>☐ I have more bloating<br>☐ I have more cramping pains<br>☐ I have more issues with flatulence<br>☐ No symptoms<br>☐ Other (please specify)
Which other symptoms do you have with wheat or gluten?	__________________________________
Does soya cause you symptoms?	◯ Yes<br>◯ No<br>◯ Not sure as I have never tried this
Which symptoms do you find are worse when you have soya (tick all that apply)?	☐ I pass stool more often<br>☐ My stools are more liquid<br>☐ I pass stool less often<br>☐ My stools are more hard<br>☐ I have more problems with soiling (staining in the underwear)<br>☐ I have more accidents with stools<br>☐ I have more bloating<br>☐ I have more cramping pains<br>☐ I have more issues with flatulence<br>☐ No symptoms<br>☐ Other (please specify)
Which other symptoms do you have with soya?	__________________________________
Do legumes or pulses (i.e. beans, lentils and peas) cause you symptoms?	◯ Yes<br>◯ No<br>◯ Not sure as I have never tried these
Which symptoms do you find are worse when you have legumes or pulses (tick all that apply)?	☐ I pass stool more often<br>☐ My stools are more liquid<br>☐ I pass stool less often<br>☐ My stools are more hard<br>☐ I have more problems with soiling (staining in the underwear)<br>☐ I have more accidents with stools<br>☐ I have more bloating<br>☐ I have more cramping pains<br>☐ I have more issues with flatulence<br>☐ No symptoms<br>☐ Other (please specify)
Which other symptoms do you have with legumes or pulses?	__________________________________
Do fruits ever cause you symptoms?	◯ Yes<br>◯ No<br>◯ Not sure as I have never tried these

Which fruits seem to cause you symptoms?

____

Which symptoms do you find are worse when you have these fruits (tick all that apply)?

☐ I pass stool more often
☐ My stools are more liquid
☐ I pass stool less often
☐ My stools are more hard
☐ I have more problems with soiling (staining in the underwear)
☐ I have more accidents with stools
☐ I have more bloating
☐ I have more cramping pains
☐ I have more issues with flatulence
☐ No symptoms
☐ Other (please specify)

Which other symptoms do you have with these specific fruits?

____

Do onions or garlic ever cause you symptoms?

◯ Yes
◯ No
◯ Not sure as I have never tried these

Which symptoms do you find are worse when you have onions or garlic (tick all that apply)?

☐ I pass stool more often
☐ My stools are more liquid
☐ I pass stool less often
☐ My stools are more hard
☐ I have more problems with soiling (staining in the underwear)
☐ I have more accidents with stools
☐ I have more bloating
☐ I have more cramping pains
☐ I have more issues with flatulence
☐ No symptoms
☐ Other (please specify)

Which other symptoms do you have with onions or garlic?

____

Do chilli or spicy food ever cause you symptoms?

◯ Yes
◯ No
◯ Not sure as I have never tried this

Which symptoms do you find are worse when you have chilli or spicy foods (tick all that apply)?

☐ I pass stool more often
☐ My stools are more liquid
☐ I pass stool less often
☐ My stools are more hard
☐ I have more problems with soiling (staining in the underwear)
☐ I have more accidents with stools
☐ I have more bloating
☐ I have more cramping pains
☐ I have more issues with flatulence
☐ No symptoms
☐ Other (please specify)

Which other symptoms do you have with chill or spicy foods?

____

Do high sugar-containing foods cause you symptoms (i.e. sweets, chocolate or sugar-containing soda)?	◯ Yes<br>◯ No<br>◯ Not sure as I have never tried this
Which symptoms do you find are worse when you have high-sugar food or drinks (tick all that apply)?	☐ I pass stool more often<br>☐ My stools are more liquid<br>☐ I pass stool less often<br>☐ My stools are more hard<br>☐ I have more problems with soiling (staining in the underwear)<br>☐ I have more accidents with stools<br>☐ I have more bloating<br>☐ I have more cramping pains<br>☐ I have more issues with flatulence<br>☐ No symptoms<br>☐ Other (please specify)
Which other symptoms do you have with sugary food or drinks?	__________________________________
Do artificial sweeteners cause you symptoms (i.e. diet soda, sugar-free squash)?	◯ Yes<br>◯ No<br>◯ Not sure as I have never tried this
Which symptoms do you find are worse when you have artificial sweeteners (tick all that apply)?	☐ I pass stool more often<br>☐ My stools are more liquid<br>☐ I pass stool less often<br>☐ My stools are more hard<br>☐ I have more problems with soiling (staining in the underwear)<br>☐ I have more accidents with stools<br>☐ I have more bloating<br>☐ I have more cramping pains<br>☐ I have more issues with flatulence<br>☐ No symptoms<br>☐ Other (please specify)
Which other symptoms do you have with artificial sweeteners?	__________________________________
Does any other food cause you symptoms, that we have not already asked about?	◯ Yes<br>◯ No
What are the other foods that can cause you symptoms?	__________________________________
Which symptoms do you find are worse when you have these foods (tick all that apply)?	☐ I pass stool more often<br>☐ My stools are more liquid<br>☐ I pass stool less often<br>☐ My stools are more hard<br>☐ I have more problems with soiling (staining in the underwear)<br>☐ I have more accidents with stools<br>☐ I have more bloating<br>☐ I have more cramping pains<br>☐ I have more issues with flatulence<br>☐ No symptoms<br>☐ Other (please specify)

Which other symptoms do you have with these other foods?	__________________________________
Do you currently take probiotics?	◯ Yes<br>◯ No
Could you specify which probiotic you take?	__________________________________
Is there anything else about your diet that you think we should know about?	____
